# Ciprofloxacin-Induced Antibacterial Activity Is Atteneuated by Pretreatment with Antioxidant Agents

**DOI:** 10.3390/pathogens5010028

**Published:** 2016-03-09

**Authors:** Majed M. Masadeh, Karem H. Alzoubi, Sayer I. Al-azzam, Omar F. Khabour, Ahlam M. Al-buhairan

**Affiliations:** 1 Department of Pharmaceutical Technology, Jordan University of Science and Technology, Irbid 22110, Jordan; 2 Department of Clinical Pharmacy, Jordan University of Science and Technology, Irbid 22110, Jordan; khalzoubi@just.edu.jo (K.H.A.); Salazzam@just.edu.jo (S.I.A.); 3 Department of Medical Laboratory Sciences, Jordan University of Science and Technology, Irbid 22110, Jordan; khabour@just.edu.jo; 4 College of applied Medical Sciences, King Saud University, Riyadh 12372, Saudi Arabia; buhairan@yahoo.co.uk

**Keywords:** ciprofloxacin, tempol, melatonin, pentoxifylline, antimicrobial susceptibility, MIC

## Abstract

Ciprofloxacin works through interfering with replication and transcription of bacterial DNA, which leads to increased oxidative stress, and death of bacterial cells. Drugs with strong antioxidant such as tempol, melatonin and pentoxifylline might interfere with the antibacterial activity of ciprofloxacin. In the current study, the effect of these drugs on the cytotoxicity of ciprofloxacin was investigated against several reference bacteria. Standard bacterial strains included *Escherichia coli* ATCC 35218, *Staphylococcus aureus* ATCC29213, *Pseudomonas aeruginosa* ATCC 9027, *Staphylococcus epidermidis* ATCC 12228, *Acinetobacter baumannii* ATCC 17978, *Proteus mirabilis* ATCC 12459, *Klebsiella pneumoniae* ATCC 13883, methicillin-resistant *Staphylococcus aureus* (MRSA) (ATCC 43300), and *Streptococcus pneumoniae* (ATCC 25923). The antibacterial activity of ciprofloxacin with or without treatment of bacterial cells by tempol, melatonin or pentoxifylline was assessed using the disc diffusion method and by measuring the minimum inhibitory concentration (MIC) and zones of inhibition of bacterial growth. All of the tested bacterial strains were sensitive to ciprofloxacin. When treated with tempol, melatonin or pentoxifylline, all bacterial strains showed significantly smaller zones of inhibition and larger MIC values compared ciprofloxacin alone. In correlation, reactive oxygen species (ROS) generation induced by ciprofloxacin antibacterial action was diminished by treatment of bacterial cells with tempol, melatonin or pentoxifylline. In conclusion, results indicate the possible antagonistic properties for agents with antioxidant properties such as tempol, melatonin and pentoxifylline when they are used concurrently with flouroquinolones. This could be related to the ability of these agents to inhibit oxidative stress in bacterial cells.

## 1. Introduction

Ciprofloxacin antibiotic is active against Gram-positive and Gram-negative bacteria. It is commonly used in the treatment of various infections such as urinary tract infections, chronic bacterial prostatitis, and acute uncomplicated cystitis [1]. Ciprofloxacin mechanism of action is not totally understood, yet, it starts by interfering with bacterial DNA replication and transcription through inhibition of DNA gyrase/topoisomerase II and DNA topoisomerase IV [2]. This eventually leads to the formation of quinolone-enzyme-DNA complexes, and thus, the generation of oxidative free radicals as singlet oxygen (^1^O_2_) and superoxide anion (O_2_^−^) [3], and the subsequent cellular death [4,5]. In addition, multiple of side effects of ciprofloxacin including phototoxicity and tendinopathies were correlated with ROS generation [3,6]. Finally, failure of ciprofloxacin treatment was reported in elderly patients taking supplement preparations [7].

Melatonin (N-acetyl-5-methoxytryptamine) is a naturally occurring antioxidant compound that is found in animals, plants, and microbes. It is released from the pineal gland during dark periods, and is suppressed upon exposure to day light [8]. Melatonin reduces the formation of free radicals either by direct scavenging of these anions (e.g., superoxide anions, hydroxyl radical, hydrogen peroxide, nitric oxide, singlet oxygen, *etc*.) or by increasing the antioxidant activity of glutathione-S transferase and glutathione reductase enzymes [9]. Tempol (4-hydroxy-2,2,6,6-tetramethylpiperidine-N-oxyl) is an antioxidant that works as a superoxide dismutase mimetic [10]. It directly reacts with both carbon-centered and peroxy radicals [11] and thus prevents the reduction of hydrogen peroxide to the hydroxyl radical [10]. Pentoxifylline (PTX), a well-tolerated methylxanthine and phosphodiesterase inhibitor has a potent antioxidant activity [12,13,14,15].

Given that ciprofloxacin works by induction of oxidative damage in bacteria [16,17], it is possible that agents with antioxidant activity could also attenuates the antibacterial activity of ciprofloxacin. In support of that, vitamins E and C [18], and vitamin B12 were shown to attenuate the bacterial activity of ciprofloxacin [19]. As a conformation of this principle, a number of antioxidant agents including tempol, melatonin or PTX were tested for possible interference with ciprofloxacin antibacterial activity. These drugs were selected based on their potential activation of endogenous antioxidant mechanism, which is different from the action of vitamins. The results of this study could be of clinical significance due to the common use of antioxidant agents along with ciprofloxacin.

## 2. Results

In this work, the possible interactive effect of tempol, melatonin or pentoxifylline with ciprofloxacin antibacterial activity was investigated against different species of reference bacteria, namely, *E. coli*, *S. aureus*, *P. aeruginosa*, *S. epidermidis*, *A. baumannii*, *P. mirabilis*, and *K. pneumoniae*. The results shown in Table 1 revealed that ciprofloxacin possessed antibacterial activity against tested reference bacteria. A zone of inhibition of 15 mm was selected to represent susceptibility of bacteria to each drug. When bacteria were treated with combination of ciprofloxacin and tempol, melatonin or pentoxifylline, the zones of inhibition of the combination were significantly lower than those of ciprofloxacin alone for all tested bacterial strains (Table 1).

Next, the minimal inhibitory concentrations of ciprofloxacin alone and in combination with tempol, melatonin or pentoxifylline were measured. As shown in Table 2, treatment of various reference bacteria cells with tempol, melatonin or pentoxifylline largely inhibited antibacterial activity of ciprofloxacin alone. This is indicated by significantly higher MIC values (Table 2) for the combination of any of tempol, melatonin or pentoxifylline with ciprofloxacin as compared to ciprofloxacin alone.

**Figure 1:**
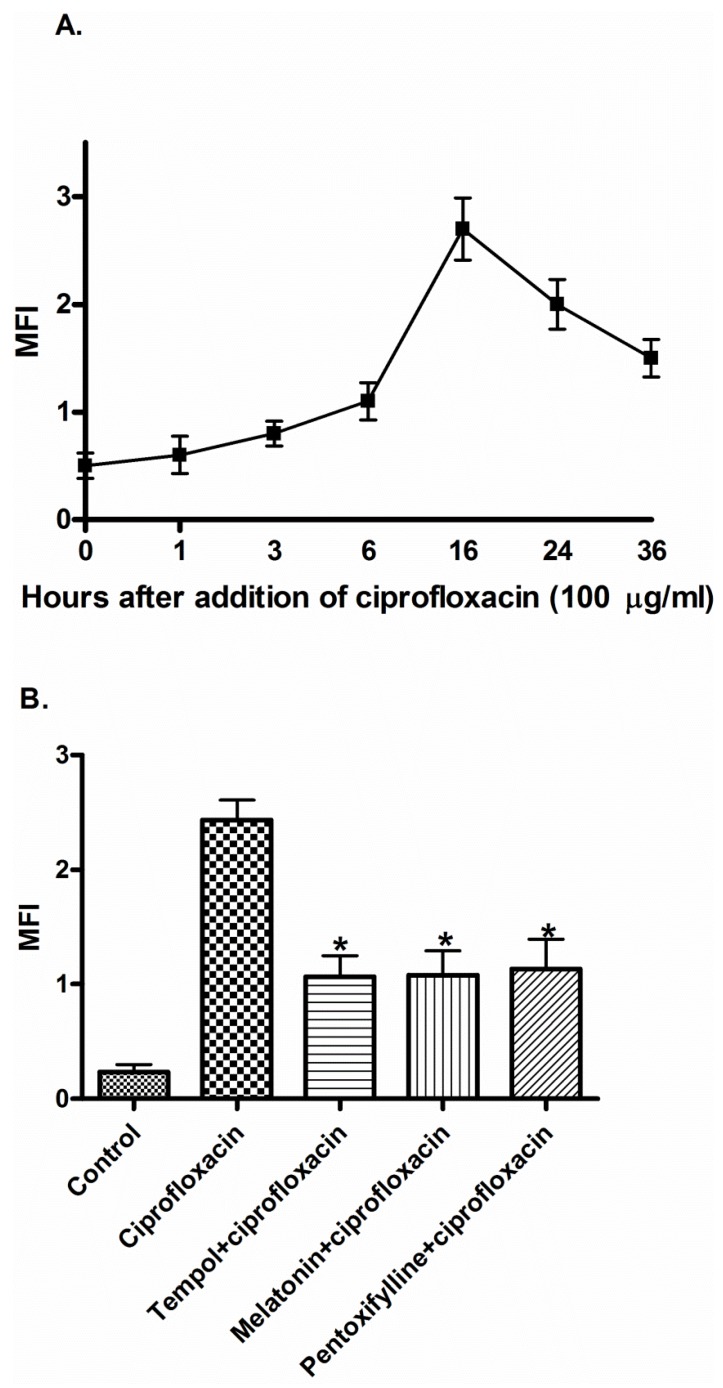
Ciprofloxacin-induced antibacterial action on *E. coli* cells is preceded by a time-dependent reactive oxygen species (ROS) generation. Figure 1 (**A**): Mean fluorescence intensity (MFI) was shown as the ratio of geometric mean fluorescence intensity of the test sample and the corresponding control. The data shown are representative of three individual experiments. Figure 1 (**B**): Pretreatment for 16 hour of *E. coli* cells with tempol, melatonin or pentoxifylline (100 µM) inhibited ciprofloxacin-induced ROS generation. 2’,7’-dichlorofluorescein diacetate (DCF-DA) (10 µM) was added for the last 30 minutes of incubation. The intensity of DCF-DA fluorescence was determined using flowcytometry with an excitation wavelength of 480 nm and an emission wavelength of 530 nm. The data shown are representative of three individual experiments. * indicates significant difference from the control, and ciprofloxacin only treated groups (One way ANOVA followed by Tukey’s post-hoc test, *p* < 0.05 in each case).

Previous work showed that induction of antibacterial activity of ciprofloxacin was via ROS generation [3,16,17]. To study this possibility, ciprofloxacin at 100 μg/mL was used to treat *E. coli* cells for various time points. Using fluorescent probe 2’,7’-dichlorofluorescein diacetate (DCFH-DA), ciprofloxacin induced an increase in ROS generation of treated cells that reached maximal level at 16 hours (Figure 1A). *E. coli* cells pretreatment with tempol, melatonin or pentoxifylline at 100 µM greatly prevented ROS generation induced by ciprofloxacin (Figure 1B). Similarly, *E.coli* cells pretreatment with tempol, melatonin or pentoxifylline at 100 µM significantly prevented cytotoxicity induced by ciprofloxacin (Table 1 and Table 2).

## 3. Discussion

This study shows that the antibacterial activity of ciprofloxacin was inhibited by the pretreatment of bacteria with antioxidants agents such as tempol, melatonin or pentoxifylline. These results were generated using wide range of standard bacterial strains. These results could be of importance when ciprofloxacin is used for bacterial infections in patients who are taking antioxidant supplements or drugs with antioxidative activity.

The results of the current study indicate that concurrent use of ciprofloxacin along with antioxidant agents such as tempol, melatonin or pentoxifylline resulted in inhibition of the antibacterial activity of ciprofloxacin against a panel of reference bacterial strains. To our knowledge, this is the first report of such effect or drug-drug interaction. Results thus could point out that simultaneous ciprofloxacin use along with antioxidant supplements might negatively interact with the antibacterial activity of ciprofloxacin. Therefore, the use of antioxidant supplements or drugs might need to be monitored in patients who are taking ciprofloxacin.

The mechanism for this interactive effect of ciprofloxacin and antioxidant supplements, namely, tempol, melatonin or pentoxifylline is unknown. The bactericidal action of ciprofloxacin is exerted by inhibition of bacterial DNA gyrase, type II topoisomorase, eventually leading to ROS generation and bacterial cell death [20,21,22,23]. Current results showed that the cytotoxicity of ciprofloxacin against bacterial cells was associated with a time-dependent ROS generation. This generation of ROS was attenuated via treatment of bacterial cells with antioxidant agents including tempol, melatonin or pentoxifylline. These results are in accordance with our previous reports with other ROS scavengers, namely vitamin C and vitamin E [18].

This study has some limirtations. For example, concentration-effect should be performed for each of the antioxidants under study. Yet, a concentration effect study will be carried out as part of our future work. We also considered the ciprofloxacin alone as positive control. However, we did not use a negative control. This *in vitro* study was aimed to prove the concept of interference form common antioxidants such as tempol, melatonin, and pentoxifylline with the antibacterial activity of ciprofloxacin. Thus, the concentrations of antioxidants used are higher than ones normally used in clinical practice. Further work with lower concentration and using human subjects are future directions.

## 4. Materials and Methods

### 4.1. Chemicals

Ciprofloxacin used in this study was donated by Al-Hikma Pharmaceuticals (Amman, Jordan). Tempol, melatonin, and pentoxifylline were obtained from Sigma-Aldrich Corporation (St Louis, Mo, USA). All drugs were used as raw material.

### 4.2. Microbial Culture and Growth Conditions

Antibacterial activity of combinations of ciprofloxacin with tempol, melatonin or pentoxifylline were evaluated against different reference bacteria including *Escherichia coli* ATCC 35218, *Staphylococcus aureus* ATCC29213, *Pseudomonas aeruginosa* ATCC 9027, *Staphylococcus epidermidis* ATCC 12228, *Acinetobacter baumannii* ATCC 17978, *Proteus mirabilis* ATCC 12459, *Klebsiella pneumoniae* ATCC 13883, methicillin-resistant *Staphylococcus aureus* (MRSA) (ATCC 43300), and *Streptococcus pneumoniae* (ATCC 25923). The organisms were stored at −70 °C in trypticase-soy broth and 20% glycerol (Becton Dickinson, East Rutherford, NJ, USA). When ready for batch susceptibility testing, samples were thawed. Minimum inhibitory concentrations (MICs) were determined in accordance with the Clinical and Laboratory Standards Institute [24].

### 4.3. Antimicrobial Susceptibility Test

Antibiotic solutions were prepared on the day of use according to the manufacturer’s recommendations. A wide range of ciprofloxacin concentrations were tested against different organisms. Serial 2 fold dilutions were added to molten BBL Muller-Hinton Gold II agar from BBL Microbiology Systems. After slight cooling and drying of the plates, a steer replicator was used to place aliquots containing approximately 5 × 10^4^ colony forming units per 50 µL for each tested bacterial strain. The plates were incubated at 37 °C and read 24 hours later. In each experiments, ciprofloxacin (100 µg/mL) alone or in combination with a final concentration of 100 µM of tempol, melatonin or pentoxifylline were added to agar right before they were added to plates for 24 hrs incubation period [25,26,27]. Results were recorded (mean of three independent experiments) by measuring the zones of growth inhibition surrounding the antibiotic containing discs.

### 4.4. Determination of Minimum Inhibitory Concentration (MIC)

The MIC was determined by serial dilution method according to the National Committee for Clinical Laboratory Standards [24]. Briefly, drugs were serially diluted, and added to plates containing molten BBL Muller-Hinton Gold II agar (Becton Dickinson, Franklin Lakes, NJ, USA). Thereafter, plates were slightly cooled and dried. Then, aliquots containing about 5 × 10^4^ colony forming units per drop of different bacterial strains were placed in each plate using an a steer replicator. Plates were read after an 18-hour incubation period at 37 °C. MIC is defined as the lowest concentration at which no growth, a faint haze or fewer than three discrete colonies were detected. Plates were read in duplicate, and the highest MIC values were recorded. The breakpoints indicated in the tables of the National Committee for CLSI [24], were used to determine susceptibility and resistance. Tests were repeated three times.

### 4.5. Measurement of ROS Generation

ROS generation was followed up via measuring generation of hydrogen peroxide. *E. coli* bacterial cells were cultured using nutrient broth (Hi-media, M002) and were treated with ciprofloxacin (100 µg/mL) for variable points of time. *E. coli* bacterial cells were then incubated with the fluorescent probe 2’,7’-dichlorofluorescein diacetate (DCF-DA) from Sigma Aldrich (Carlsbad, CA, USA) for 30 minutes. The intensity of DCF-DA fluorescence was determined by using a FACS can flow cytometer (Becton Dickinson, Franklin Lakes, NJ, USA), with an excitation wavelength of 480 nm and an emission wavelength of 530 nm.

### 4.6. Statistical Analysis

Analysis was performed using GraphPad Prism software (version 4.0). One-way ANOVA followed by Tukey’s post-test was used to determine if there is a statistically significant difference. *p*-values < 0.05 were considered significant.

## 5. Conclusions

The antibacterial action of ciprofloxacin is attenuated when they are combined with antioxidant agents. This is likely to be related to the interference with induction of ROS by ciprofloxacinby. The significance of this observation comes from the wide use of quinolones antibiotic and their great therapeutic value. Thus, investigations of the clinical consequences of simultaneous use of flourquinolones antibiotics along with antioxidant agents in patients being treated against bacterial infections are recommended.

## Figures and Tables

**Table 1:** A comparison between the zones of inhibition (mm) of ciprofloxacin (100 µg/mL) alone and ciprofloxacin in the presence of 100 µM of tempol, melatonin or pentoxifylline against standard bacterial strains.

Standard Bacterial Strains	Zone of Inhibition (mm)*						
	Ciprofloxacin	Tempol	Ciprofloxacin + Tempol	Melatonin	Ciprofloxacin + Melatonin	Pentoxifylline	Ciprofloxacin + Pentoxifylline
Gram +ve:							
*S. aureus*	22.7 ± 1.5	2.7 ± 0.6	8.7 ± 0.6	5.0 ± 1.2	12.0 ± 1.0	5. 3 ± 0.6	13. 3 ± 0.6
*S. epidermidis*	22.0 ± 1.0	3.3 ± 0.6	8.3 ± 0.6	2.7 ± 0.6	9.7 ± 0.6	2. 4 ± 0.6	12. 3 ± 0.6
*MRSA*	10.7 ± 1.5	2.7 ± 0.6	2.7 ± 0.6	3.7 ± 1.2	5.0 ± 1.0	1.2 ± 0.6	4.0 ± 1.0
*S. pneumoniae*	14.7 ± 0.6	4.3 ± 1.2	6.7 ± 0.6	4.7 ± 1.6	7.3 ± 0.6	4.7 ± 0.6	7.7 ± 0.6
*VRE*	16.7 ± 1.5	1.2 ± 0.6	1.7 ± 0.6	2.3 ± 1.2	2.3 ± 1.2	2.1 ± 1.6	3.0 ± 1.0
*S. pyogenes*	21.7 ± 1.5	4.3 ± 1.2	7.3 ± 1.2	4.7 ± 1.2	9.7 ± 0.6	4.7 ± 1.2	12.0 ± 1.0
Gram –ve:							
*E. coli*	26.7 ± 2.0	5 .0 ± 2.0	8 .0 ± 1.0	5.0 ± 2.0	13.0 ± 1.0	2.7 ± 0.6	15.7 ± 0.6
*P. aeruginosa*	23.3 ± 1.2	2.7 ± 1.6	9.7 ± 0.6	2.0 ± 1.0	13.0 ± 1.0	4. 3 ± 2.1	13.3 ± 1.2
*P. mirabilis*	19.7 ± 2.1	1.7 ± 1.2	7.7 ± 1.2	2.7 ± 0.0	8.0 ± 0.0	1.7 ± 0.6	10.7 ± 0.6
*K. pneumoniae*	22.0 ± 2.0	4.7 ± 0.6	7.7 ± 0.6	2.0 ± 1.2	12.0 ± 1.0	4.3 ± 0.6	13.0 ± 1.0
*A. baumannii*	11.3 ± 0.6	2.7 ± 0.6	2.7 ± 0.6	4.7 ± 1.6	3.7 ± 0.6	1.2 ± 0.6	5.3 ± 0.6

* The zones of inhibition values for ciprofloxacin alone were significantly (*p* < 0.05) lower than those of combination of ciprofloxacin with tempol, melatonin or pentoxifylline for all tested bacterial strains. Results are presented as mean ± SD of three independent experiments.

**Table 2:** A comparison between the minimum inhibitory concentrations (MIC; µg/mL) of ciprofloxacin alone and ciprofloxacin in the presence of 100 µM of tempol, melatonin or pentoxifylline against standard bacterial strains.

Standard Bacterial Strains	MIC (µg/mL)*			
	Ciprofloxacin	Ciprofloxacin + Tempol	Ciprofloxacin + Melatonin	Ciprofloxacin + Pentoxifylline
Gram +ve:				
*E. coli*	0.07 ± 0.04	80.00 ± 0.00	133.33 ± 57.73	133.33 ± 57.73
*S. aureus*	0.14 ± 0.09	106.60 ± 46.18	166.67 ± 57.73	166.67 ± 57.73
*S. epidermidis*	0.10 ± 0.04	106.60 ± 46.18	133.33 ± 57.73	133.33 ± 57.73
*MRSA*	0.49 ± 0.00	320.00 ± 0.00	666.67 ± 230.9	533.33 ± 230.90
*S. pneumonia*	0.37 ± 0.17	266.60 ± 92.370	800.00 ± 0.00	666.67 ± 230.90
*VRE*	0.99 ± 0.00	640.00 ± 0.00	133.33 ± 461.80	133.33 ± 461.80
*S. pyogenes*	0.16 ± 0.07	133.30 ± 46.18	133.33 ± 57.73	133.33 ± 57.73
Gram –ve:				
*P. aeruginosa*	0.14 ± 0.09	133.30 ± 46.18	333.33 ± 115.4	266.67 ± 115.4
*P. mirabilis*	0.21 ± 0.07	160.00 ± 0.00	266.67 ± 115.4	200.00 ± 0.00
*K. pneumonia*	0.19 ± 0.08	133.30 ± 46.18	133.33 ± 57.73	266.67 ± 115.40
*A. baumannii*	0.49 ± 0.00	426.60 ± 184.70	800.00 ± 0.00	166.67 ± 461.80

* In each experiments, ciprofloxacin (100 µg/mL) alone or in combination with a final concentration of 100 µM of tempol, melatonin or pentoxifylline were added to agar right before they were added to plates for 24 hrs incubation period. The MIC values for ciprofloxacin alone were significantly (*p* < 0.05) lower than those of combination of ciprofloxacin alone and ciprofloxacin in the presence of tempol, melatonin or pentoxifylline for all tested bacterial strains. Results are presented as mean ± SD of three independent experiments.
